# Evaluating Calcium Hydroxide Removal Techniques in Endodontics: A Comparative Analysis 

**DOI:** 10.30476/dentjods.2025.103049.2416

**Published:** 2025-12-01

**Authors:** Vahid Fallahi Sarvenoei, Mohsen Aminsobhani, Babak Farzaneh, Mohammad Ali Ketabi

**Affiliations:** 1 Dept. of Prosthodontics, School of Dentistry, Tehran University of Medical Sciences, Tehran, Iran.; 2 Dept. of Endodontics, School of Dentistry, Aja University of Medical Sciences, Tehran, Iran.

**Keywords:** Calcium Hydroxide, Endodontics, Ethylene-Diamine-Tetra-Acetic Acid, Sodium Hypochlorite, Root Canal Preparation

## Abstract

**Background:**

Endodontic therapy plays a pivotal role in dentistry, with effective removal of intracanal medications crucial for successful treatment. The lingering presence of calcium hydroxide within dentinal walls can impede sealer adhesion and compromise treatment outcomes.

**Purpose:**

This study aimed to compare the efficacy of various methods for removing calcium hydroxide from different regions of the root canal wall.

**Materials and Method:**

In this *in vitro* randomized trial study, 108 extracted teeth underwent canal cleaning and shaping using the Dentsply Protaper Gold rotary system. Subsequently, except for the negative control group, all teeth were filled with calcium hydroxide and divided into eight groups. These groups underwent different cleaning protocols involving Gentle Brush or Gentlefile #021 files or master apical file in combination with ethylene-diamine-tetra-acetic acid or sodium hypochlorite solutions. After tooth splitting, stereomicroscopic images were taken, and Digimizer software was utilized to calculate residual calcium levels in coronal, middle, and apical regions. Mann-Whitney test was used to check the effect of the cleaning method and type of washing solution among the methods employed. All the analyses were conducted using SPSS 22.

**Results:**

The results indicated that the Gentle Brush method's superior efficacy in calcium hydroxide removal compared to other files, which was statistically significant (*p* Value <0.01). Similarly, the ethylene-diamine-tetra-acetic acid rinse solution proved more effective than sodium hypochlorite in clearing calcium hydroxide from the canal wall (*p*< 0.05).

**Conclusion:**

The findings suggest that a Gentle Brush combined with an ethylene-diamine-tetra-acetic acid washing solution represents the most effective method for canal cleaning and calcium hydroxide removal. This study underscores the importance of employing efficient techniques to enhance treatment quality in endodontic practice.

## Introduction

Endodontic therapy is a cornerstone in dentistry and is pivotal in ensuring the success of dental treatments. Endodontic therapy, also known as root canal treatment, aims to alleviate pain and preserve natural teeth by treating infections and inflammation within the tooth's pulp chamber and root canals [ 1 ]. A key determinant of this success lies in effectively eradicating microorganisms within the root canal system [ 2 - 3 ]. To achieve this, using intracanal medicaments for disinfection is a widespread practice to enhance treatment outcomes [ 4 ]. Among these medicaments, calcium hydroxide is favored for its antimicrobial properties and ability to deter root resorption [ 5 - 6 ].

Complete removal of intracanal medicaments, particularly Ca(OH)_2_, remains a critical challenge in endodontic practice . Residual Ca(OH) 2 within dentinal walls can compromise treatment quality by obstructing sealer adhesion, promoting apical liquefaction, and interfering with the bond between filling materials and dentinal tubules through the formation of calcium eugenol [ 9 ]. These residues not only reduce the sealing ability of root canal fillings but also create unfavorable conditions for tissue healing, potentially leading to delayed periapical repair and persistent symptoms [ 10 - 12 ]. Systematic reviews emphasize the technical difficulty of removing Ca(OH)_2_, highlighting the need for advanced irrigation techniques to ensure thorough debridement [ 13 - 14 ]. These insights underscore the importance of meticulous intracanal cleaning to enhance clinical outcomes in endodontic treatment [ 15 - 16 ]. 

Various techniques have been developed to optimize this process. Among these, the most commonly employed method involves recapitulation with a master apical file (MAF) in combination with copious irrigation, which remains a fundamental technique for effective Ca(OH)_2_ removal [ 17 - 19 ]. More advanced methods, such as passive ultrasonic irrigation and laser-activated irrigation, have demonstrated significantly higher efficacy in removing Ca(OH)_2_ [ 20 - 21 ]. Additionally, sonic activation techniques like EDDY have also been shown to be effective, achieving comparable results to passive ultrasonic irrigation. Other methods include chelating agents like ethylenediaminetetraacetic acid (EDTA) and sodium hypochlorite (NaOCl), which enhance the chemical dissolution of Ca(OH)_2_ when used in conjunction with mechanical agitation [ 22 - 23 ]. 

Despite advancements in endodontic techniques, studies have demonstrated that using EDTA as a standalone irrigation method is often insufficient for effectively removing Ca(OH)_2_ from root canals. While EDTA is effective at chelating inorganic materials, its use alone may not achieve optimal cleaning results. Consequently, it is recommended to employ a combination of techniques, such as using NaOCl or EDTA in conjunction with recapitulation using the MAF or employing ultrasonic activation. These methods enhance the mechanical agitation and chemical efficacy needed to thoroughly clear residual Ca(OH)_2_ from the canal walls. The ongoing search for a definitive solution underscores the imperative to improve treatment efficacy and patient outcomes, highlighting the need for innovative irrigation protocols that integrate multiple strategies to ensure comprehensive cleaning of the root canal system [ 24 - 25 ]. This pursuit is essential not only for effective disinfection but also for enhancing the overall success of endodontic therapy [ 16 , 26 ].

In light of the challenges mentioned above, the present study aims to evaluate the efficacy of Gentle Brush and Gentlefile #021 compared to standard washing methods for removing Ca(OH)_2_ from various regions of the root canal wall. Gentlefile, with the respective handpiece operating at 6500 rpm, has a unique shape and special structure that can match the wall of different channels. By meticulously assessing these instruments using a stereomicroscope, we aimed to provide insights into their effectiveness in achieving thorough canal cleaning and rinsing [ 27 ]. Ultimately, this manuscript sought to contribute to the ongoing discourse on optimizing endodontic treatment protocols to enhance treatment quality and patient satisfaction [ 28 ]. 

## Materials and Method

### Study design

This research was approved by the Ethics Committee of the Department of Endodontics, School of Dentistry, Aja University of Medical Sciences, Tehran, Iran, under the identification number IR.AJAUMS.REC.1399.079. The study was designed as an *in vitro* randomized trial, utilizing 108 single-canal central and lateral teeth extracted for orthodontic or periodontal reasons. The inclusion criteria were strictly defined to ensure sample homogeneity among samples. They included single-canal morphology, absence of both internal and external pathology, no history of prior root canal treatment, absence of visible fractures or cracks, lack of calcification within the canal, complete apical root development, and the presence of severe root curvature.

Standardized periapical radiographs were obtained at mesial and distal angles to confirm these criteria, following established radiographic protocols. These radiographs were used to evaluate root morphology apical formation and to detect any potential fractures or calcifications [ 29 ]. Teeth were ethically sourced from dental clinics in Kermanshah, and proper consent and compliance with ethical guidelines for biological specimen handling were ensured.

### Sample preparation

Severe curvature (>30°) was excluded to reduce confounding variables, ensuring uniformity among samples [ 29 - 30 ]. Additionally, the crowns of all teeth were sectioned at the cementoenamel junction (CEJ) using a diamond disk (Bego, Berman, Germany) to standardize the working length across specimens [ 31 ]. Surface disinfection was performed by immersing the teeth in a 5.25% NaOCl solution for 30 minutes, followed by storage in a physiological saline solution at room temperature to preserve sample integrity. The working length was determined with #10 K-file (Dentsply Maillefer, Ballaigues, Switzerland) and established at 1mm shorter than the apical foramen [ 32 ]. 

For canal preparation, a glide path was first created manually, followed by rotary file shaping using the Protaper system (Dentsply Sirona, NitiGold, Switzerland) up to the F3 file, as per manufacturer guidelines [ 32 ]. Throughout instrumentation, irrigation with 5ml of 5.25% NaOCl (Cerkamed, Stalowa Wola, Poland) was utilized to ensure proper cleaning [ 33 ]. This standardized approach to canal preparation, irrespective of the initial canal diameter, enabled a consistent baseline for comparing the efficacy of Ca(OH)_2_ removal techniques while minimizing the influence of anatomical variations.

### Sample size

The sample size for this study was determined based on a prior study [ 34 ], assuming an estimated variance of 1.5 and a minimum detectable difference of 0.5. A confidence level of 95% and statistical power of 80% were applied to ensure robustness. The calculation resulted in approximately 17 samples per group. With six groups of 17 samples each and two control groups of three samples each, the total sample size was set at 102.

### Experimental Protocol

There were six groups of 17 samples and two control groups of three. All specimens were enveloped in hot wax following canal preparation to simulate periapical tissue (Cavex modeling wax, Holland BV, Haarlem, Netherlands) [ 35 ]. Ca(OH)_2_ was then uniformly filled into the canals using Lentulo spirals, ensuring comprehensive filling by successive compaction with a #80 plugging instrument. Ca(OH)_2_ used in this study was a premixed formulation (Golchai, Iran). The premixed nature ensured uniform consistency and ease of application during canal filling. The material was delivered into the canals using Lentulo spirals to achieve homogeneous distribution across all regions of the canal. Premixed Ca(OH)_2_ was chosen to minimize variability associated with manual preparation and ensure reproducibility of results in future studies. This standardized approach facilitates the replication of our methodology by researchers aiming to evaluate similar techniques for Ca(OH)_2_ removal. Subsequently, a 3mm diameter Cavit™ dressing (3M ESPE, Seefeld, Germany) was used to seal the coronal portion of the canal completely. 

To ensure the integrity of the coronal section and eliminate the potential influence of the temporary filling material (Cavizol), all temporary fillings were meticulously removed using a diamond burr under magnification before analysis. This step was performed uniformly across all groups to standardize the canal conditions and minimize any potential bias in the results for the coronal section. Additionally, stereo microscopy and Digimizer software were employed to quantify residual Ca(OH)_2_ only after ensuring the complete removal of temporary fillings and associated debris. This approach aimed to ensure that the results obtained for the coronal section accurately reflect the efficacy of cleaning techniques rather than artifacts introduced by the temporary material. Radiographic confirmation of complete filling was obtained, and any incompletely filled samples underwent refilling [ 36 ].

**Figure 1:**
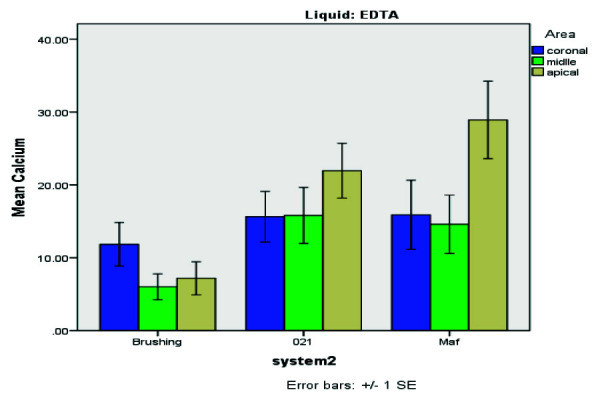
Percentage of residual Ca(OH)_2_ in different groups with ethylene-diaminetetra-acetic acid (EDTA) rinsing solution

**Figure 2:**
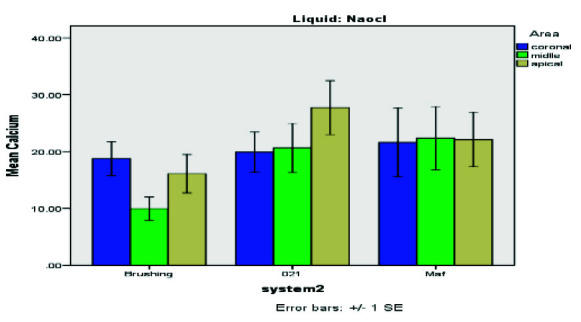
Percentage of residual Ca(OH)_2_ in different groups subjected to NaOCl rinsing solution

Following a ten-day incubation period at 37°C in physiological saline solution, as per established protocols, teeth were thoroughly cleaned with temporary dressing. The samples were then randomly divided into six groups of 17 cases (Figures 1-2). Two positive and negative control groups (three in each group) were divided into six experimental groups of 17 cases each, alongside positive and negative control groups (three in each group), to facilitate subsequent comparative analysis. The experimental groups were delineated as follows:

**Group A:** Seventeen teeth were subjected to Gentle Brush irrigation combined with 4 ml of EDTA solution per tooth for Ca(OH)_2_ removal. Following dressing removal, a glide path was established in the canal using the MAF file, tailored to each tooth's specific operating length. Subsequently, 2ml of EDTA was injected into the canal, followed by a final rinse with an additional 2ml of EDTA. 

**Group B:** In this cohort, 17 teeth underwent irrigation with Gentle Brush accompanied by 4ml of NaOCl. After using each file, the flutes were cleaned with gauze dipped in alcohol. First, a #10 K-file was used to the working length to ensure apical patency. Next, a Gentle Brush was used with a pecking motion and gentle pressure for 5 seconds to reach the apical third of the canal. After reaching the working length, a final rinse was performed. Similar to Group A, dressing removal was followed by glide path creation and irrigation with 2ml of NaOCl. One-minute irrigation was performed using Gentle Brush, followed by a final rinse with 2ml of EDTA.

**Group C:** Consisting of 17 teeth, this group was subjected to Gentlefile #021 irrigation coupled with EDTA solution for Ca(OH)_2_ removal. Following dressing removal, a glide path was created using the relevant MAF file, followed by injection of 2ml of EDTA into the canal, with an additional 2ml of EDTA used for final rinsing.

**Group D:** Similar to Group C, 17 teeth underwent irrigation with Gentlefile #021 paired with NaOCl. After dressing removal, a glide path was established, and 2 ml of NaOCl was injected into the canal. Gentlefile #021 was utilized for one-minute irrigation, followed by a final rinse with 2 ml of NaOCl.

**Group E:** Seventeen teeth received irrigation with MAF and EDTA solution. Following dressing removal, a glide path was created using the relevant MAF file, and 2ml of EDTA solution was injected into the canal, followed by a final rinse with an additional 2ml of EDTA.

**Group F:** In this cohort, dressing removal was followed by glide path creation using the relevant MAF file for each of the 17 teeth. Subsequently, 2 ml of NaOCl was injected into the canal, followed by a final rinse with 2 ml of NaOCl.

**Group G (Positive Control):** This group was included to serve as a baseline for comparison, representing conditions where Ca(OH)_2_ remained fully retained within the canal. Teeth in this group were intentionally prepared without subsequent irrigation or cleaning protocols, ensuring the presence of Ca(OH)_2_ throughout the coronal, middle, and apical regions. This configuration provided a reference point for evaluating the effectiveness of different cleaning techniques applied in the experimental groups.

**Figure 3:**
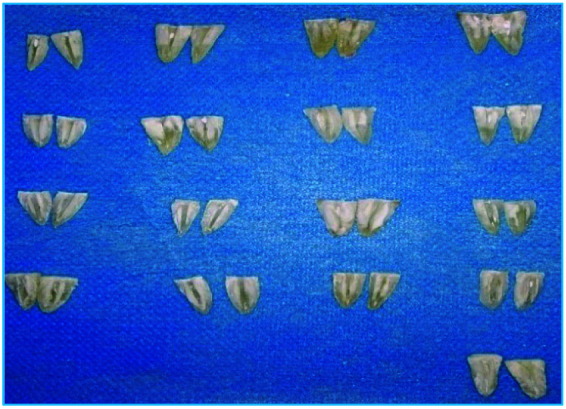
Broken teeth with chisels

**Group H (Negative Control):** The negative control group comprised teeth devoid of Ca(OH)_2_. After completing the procedures above, a decision was made to utilize a chisel for tooth splitting rather than a cutting method. This decision was based on the need for the disc to effectively remove Ca(OH)_2_ while maintaining the integrity of the canal surface during the subsequent fracture steps [ 37 ]. To facilitate this process, a diamond fissure bur 008 (D&Z, Germany) was employed to create a groove around the tooth, aiming to achieve a more controlled and efficient halving with reduced energy expenditure. This approach was adopted to mitigate potential issues such as incomplete tooth breakage, thereby ensuring optimal experimental outcomes (Figure 3) [ 38 ].

**Figure 4:**
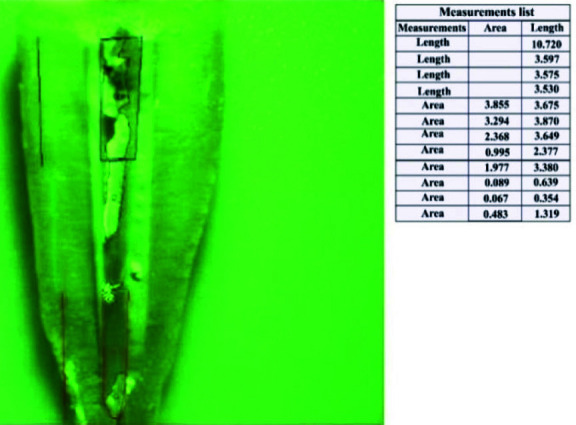
The user interface of Digimizer software used for the analysis and quantification of Ca(OH)_2_ levels in root canal specimens

After the specimens were individually fragmented, stereo microscopy, a specialized ocular instrument capable of providing three-dimensional visualization, was employed. This microscopy technique allows for captureng images at appropriate magnifications, facilitating detailed examination. Images of each tooth half were captured at a magnification of 1× (10 times). Subsequently, Digimizer software version 4.5 was utilized to analyze these images and quantify the levels of Ca(OH)_2_ present in each tooth. The software delineated different regions within the images, including the coronal, middle, and apical levels, distinguishing areas containing Ca(OH)_2_ from calcium-free surfaces. The software then computed the surface area of these regions in square millimeters (mm^2^) (Figure 4 for an illustration of the Digimizer software interface).

### Statistical Analysis

The data from each group were entered into Microsoft Excel 2019. Ca(OH)_2_ levels for each sample were calculated as percentages relative to the total level, and group averages were computed. Statistical analysis was performed using SPSS 22, with the Kolmogorov-Smirnov test used to assess data distribution and the Kruskal-Wallis test employed for intergroup comparisons.

## Results

### Quantification of Residual Ca(OH)_2_

The quantification of residual Ca(OH)_2_ was performed by a blinded evaluator. Figure 1 summarizes the mean percentage of residual Ca(OH)_2_ for each group treated with EDTA, displaying residual levels across experimental groups. Figure 2 presents the residual Ca(OH)_2_ levels for the NaOCl groups. The positive control group (Group 7) retained 100% Ca(OH)_2_, while the negative control group (Group 8) had no residual calcium. The Kolmogorov-Smirnov test (*p*< 0.001) confirmed a non-normal data distribution, leading to the use of the Kruskal-Wallis test for intergroup comparisons.

### Comparative Efficacy of Cleaning Systems

Statistical analysis identified significant differences in residual Ca(OH)_2_ levels among irrigation techniques and specific root canal regions. Gentle Brush exhibited significantly lower residual Ca(OH)_2_ levels in the middle region compared to Gentlefile #021 (*p*= 0.021). In the apical region, Gentle Brush also showed significantly greater Ca(OH)_2_ removal efficacy than Gentlefile #021 (*p*= 0.001) and MAF (*p*= 0.008).

**Figure 5:**
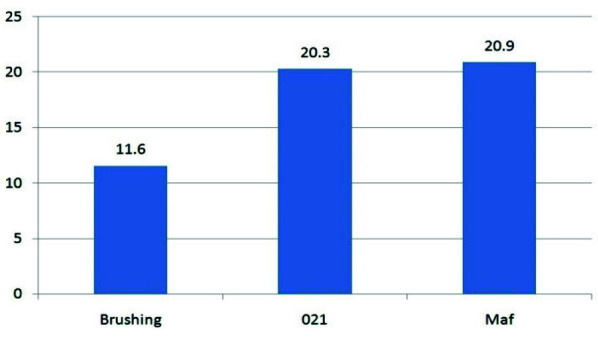
Comparison of average residual Ca(OH)_2_ levels following root canal cleansing using different files (Gentle Brush, MAF, Gentlefile #021)

Furthermore, when Gentle Brush was used with EDTA, it achieved the lowest residual Ca(OH)_2_ levels in the apical region, representing the only statistically significant finding for this region. No significant differences in Ca(OH)_2_ removal were observed among the experimental groups in the coronal and middle thirds. Figure 5 presents the results of the Mann-Whitney U test, comparing residual Ca(OH)_2_ levels across the Gentle Brush, MAF, and Gentlefile #021 groups. 

### Comparison of Irrigation Solutions (EDTA vs. NaOCl)

**Figure 6:**
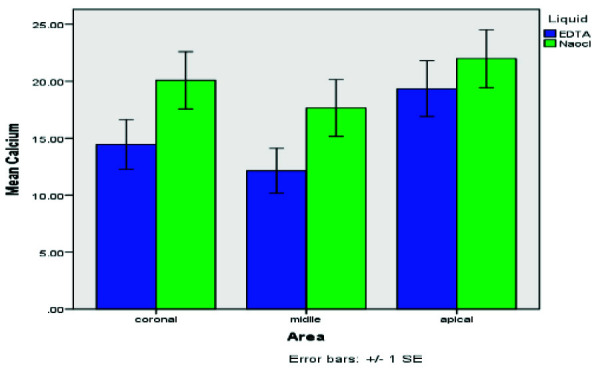
Comparison of mean residual Ca(OH)_2_ levels in different regions of the root canal by ethylene-diamine-tetra-acetic acid (EDTA) and NaOCl irrigation solutions

A statistically significant difference in residual Ca(OH)_2_ levels was observed between teeth treated with Gentlefile #021 and those treated with Gentle Brush (*p*< 0.001), with Gentle Brush demonstrating lower residual Ca(O-H)₂ levels. The Mann-Whitney U test was performed to compare the efficacy of EDTA and NaOCl in Ca(OH)₂ removal. Table 1 presents the mean residual Ca(OH)₂ levels across different root canal regions for both irrigation solutions, while Figure 6 visualizes these comparisons. According to Table 1, EDTA exhibited superior efficacy in Ca(OH)₂ removal across all root canal regions, leaving lower residual levels than NaOCl. The most significant difference was in the middle segment (12.14 vs. 17.67), while EDTA also outperformed NaO-Cl in the coronal (14.45 vs. 20.07) and apical (19.34 vs. 21.97) regions. These findings confirm EDTA’s higher efficiency, particularly in the middle segment. Figure 6 illustrates the mean residual Ca(OH)₂ levels across different regions of the root canal following irrigation with EDTA and NaOCl solutions. The results indicate that NaOCl irrigation led to higher residual calcium hydroxide levels in all three regions- coronal, middle, and apical—compared to EDTA. The difference is particularly notable in the middle and coronal regions, where EDTA demonstrated a more effective removal of Ca(OH)₂. The error bars represent ±1 standard error, highlighting the variability of the measurements. 

**Table 1:** Comparison of mean residual Ca(OH)_2_ in different regions of the root canal by ethylene-diamine-tetra-acetic acid (EDTA) and NaOCl irrigation solutions

Irrigation Solution	Standard Deviation	Mean	Cleaning Area
EDTA	22	14.45	Coronal
EDTA	19.9	12.14	Middle
EDTA	24.7	19.34	Apical
NaOCl	20	20.07	Coronal
NaOCl	25	17.67	Middle
NaOCl	25.5	21.97	Apical

### Regional Variability in Residual Ca(OH)_2_ Removal

**Figure 7:**
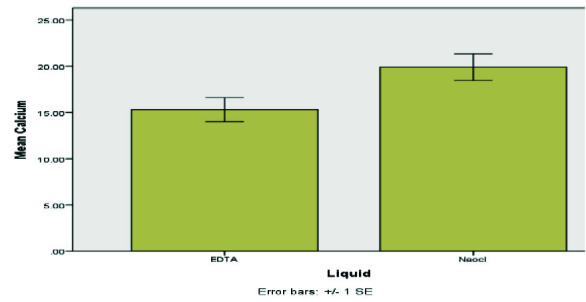
Comparison of mean residual Ca(OH)_2_ of teeth by ethylene-diamine-tetra-acetic acid (EDTA) rinses and NaOCl

Table 1 and Figures 6-7 further substantiate EDTA's superior performance across all regions. The data underscore Gentle Brush's design advantage, facilitating enhanced EDTA activation and thorough Ca(OH)_2_ removal, particularly in challenging apical regions. The EDTA rinsing solution was most effective when used with the Gentle Brush system, showing superior results in the coronal (*p*= 0.008), middle (*p*= 0.015), and apical (*p*= 0.010) regions. Residual Ca(OH)_2_ levels were highest in the apical region compared to other areas.

## Discussion

Effective root canal cleaning is fundamental for successful endodontic therapy. Ca(OH)_2_ is extensively used as an intracanal medicament due to its antimicrobial properties and tissue-dissolving capabilities, attributed to its release of hydroxyl ions in an alkaline environment [ 39 ]. However, its residues must be completely removed before obturation to ensure optimal sealer adhesion and prevent treatment failure. 

This challenge is particularly pronounced in the apical third of the canal, where the complex anatomy hinders effective debridement. Residual Ca(OH)_2_ can obstruct sealer penetration and bonding, leading to compromised treatment outcomes. Thus, employing advanced irrigation protocols is crucial to overcome these anatomical barriers and achieve thorough cleaning [ 40 - 41 ]. 

This study confirmed EDTA's superior effectiveness over NaOCl in reducing residual Ca(OH)_2_ across all root canal regions, particularly in the middle third (Table 1) [ 3 ]. EDTA's chelating properties facilitate the dissolution of calcium ions and the removal of the smear layer, which otherwise obstructs sealer adhesion and dentinal tubule penetration [ 42 ]. In contrast, NaOCl, while highly effective at dissolving organic tissue and exhibiting antimicrobial properties, lacks the chelating ability needed for efficient Ca(OH)_2_ removal. 

The middle third of the canal exhibited the greatest reduction in residual Ca(OH)_2_ with EDTA, reflecting its superior performance in this region [ 43 - 44 ]. However, both EDTA and NaOCl faced challenges in the apical region, where higher residual levels persisted due to anatomical complexity and reduced accessibility. 

EDTA’s ability to reduce dentin microhardness further enhances its capacity to disrupt Ca(OH)_2_ bonds on canal walls, emphasizing its critical role in achieving effective debridement and optimal treatment outcomes. Despite EDTA's overall superiority, the study highlights persistent challenges in the apical third, where the intricate anatomy complicates complete debris removal [ 45 ]. These findings emphasize the need for continued refinement of irrigation techniques to address this limitation.

The Gentle Brush method proved more effective in Ca(OH)_2_ removal than Gentlefile #021. Its design, featuring long strands, enables better agitation and coverage of the canal walls, particularly in the apical region, where its vortex flow activation improves cleaning efficacy [ 3 , 46 ]. Conversely, Gentlefile #021 demonstrated limitations in agitation and debris removal due to its less efficient mechanical action [ 47 ]. 

The Gentle Brush's design allows for more effective mechanical agitation within the canal, facilitating better contact with the canal walls and enhancing debris removal [ 48 ]. Studies have shown that mechanical cleaning methods often outperform manual techniques in terms of efficacy, as they can reach areas difficult for files to access [ 49 - 50 ].

The Gentle Brush method demonstrates superior interaction with irrigants, enhancing its effectiveness compared to the Gentlefile system [ 51 ]. Figures 3-6, and Table 1 illustrate the study results, particularly the enhanced removal of Ca(OH)_2_ when Gentle Brush is used with EDTA rinsing solution. Figure 3 highlights the effective cleaning achieved in the apical region due to the Gentle Brush file's unique design. The file threads open more efficiently in the apical area, aligning better with the root canal walls compared to the coronal area. This design, combined with the activation of EDTA through vortex flow, results in improved Ca(OH)_2_ removal in the apical region.

These findings are consistent with prior studies, which emphasize the limitations of traditional methods in cleaning the apical area. The Gentle Brush’s ability to overcome these limitations further validates its efficacy in challenging anatomical regions [ 52-54 ]. 

This study confirmed that EDTA was significantly more effective than NaOCl for removing Ca(OH)_2_, and observed the most notable efficacy in the middle region of the root canal [ 3 ]. While advanced techniques like ultrasonic or laser-based systems enhance cleaning, their adoption often requires specialized training and equipment, making them less feasible for routine practice due to increased cost and complexity. Additionally, the delicate nature of root canal anatomy and potential complications may lead practitioners to prefer more straightforward and conservative methods.

Figure 4 illustrates the residual Ca(OH)_2_ levels across groups treated with NaOCl, while Figure 5 compares the efficacy of different cleaning systems- Gentle Brush, MAF, and Gentlefile #021. The Gentle Brush consistently demonstrated superior performance, particularly in combination with EDTA. These findings underscore the importance of selecting effective yet accessible cleaning protocols and highlight the need for continued research to refine existing methods [ 55 ]. 

This study evaluated the efficacy of root canal cleaning systems, focusing on the MAF and the Gentlefile system. The Gentlefile system effectively removed Ca(OH)_2_ residues, consistent with findings by Gokturk *et al*. (2017), who highlighted variations in residual Ca(OH)_2_ levels across different canal regions [ 52 ]. A notable observation is the "packing effect" of the Gentle Brush, which may push Ca(OH)_2_ deeper into the canal. This effect facilitates enhanced removal in the coronal region by optimizing fluid dynamics and agitation [ 56 ].

Anatomical variations significantly influence cleaning efficacy. The coronal third, with its wider diameter and complex anatomy, tends to trap debris more effectively than the narrower, tapered apical third. This distinction allows for more efficient flushing and debris removal in the apical region. The effectiveness of irrigation solutions, such as NaOCl and EDTA, also plays a critical role, as these solutions can enhance cleaning outcomes, particularly in challenging apical regions . Additionally, prior research has demonstrated that irrigation methods yield varying levels of success in removing Ca(OH)_2_ across different canal thirds. These differences emphasize the need for tailored approaches to address the unique challenges presented by each region [ 59-60 ]. 

Our study quantified Ca(OH)_2_ surface areas, whereas Gokturk *et al*. [ 52 ] used a scoring system categorizing residual Ca(OH)_2_ as low, medium, or high and expressed results as percentages to highlight distinctions between methods. Gokturk *et al*. [ 52 ] also reported enhanced efficacy of NaOCl when activated differently, whereas our findings revealed that EDTA, particularly when paired with the Gentle Brush system, outperformed NaOCl in Ca(OH)_2_ removal [ 52 ]. 

Figure 6 highlights the comparative efficacy of EDTA and NaOCl irrigation solutions across various root canal regions. Although the Gentlefile system is designed to enhance irrigant flow and agitation, its mechanical action alone is insufficient to remove all residual Ca(OH)_2_, particularly in complex canal anatomies. Activation improves irrigant penetration but does not ensure complete debris removal, as some remnants may become trapped in irregular surfaces.

The design of Gentlefile, theoretically, provides better access to irregular canal areas, yet studies show that its improved fluid dynamics may still fall short in disrupting the bond between Ca(OH)_2_ and dentin. This limitation results in higher residual levels compared to methods like the Gentle Brush, which combines superior mechanical and fluid activation for more effective cleaning outcomes.

This study emphasizes the importance of combining effective irrigation solutions, such as EDTA, with mechanical systems like Gentle Brush to achieve optimal cleaning outcomes. The Gentle Brush outperforms Gentlefile, particularly in the apical third, where its superior design and activation capabilities enhance debris removal. These findings provide clinicians with valuable insights for selecting the most effective cleaning techniques, ultimately improving treatment success and patient satisfaction [ 61-62 ]. 

## Conclusion

This study evaluated the effectiveness of root canal cleaning methods for removing Ca(OH)_2_, a crucial aspect of successful endodontic therapy. The Gentle Brush system demonstrated superior performance, particularly in the apical region, where its design and mechanical efficiency ensured thorough cleaning. Additionally, it outperformed Gentlefile #021 in the middle region, further proving its effectiveness in complex anatomical areas. Among the irrigation solutions evaluated, EDTA was significantly more effective than NaOCl in removing Ca(OH)_2_ residues from canal walls. This effectiveness was most pronounced when EDTA was paired with the Gentle Brush system, achieving optimal cleaning in all root canal regions- coronal, middle, and apical. However, residual Ca(OH)_2_ levels remained highest in the apical region, reflecting the persistent anatomical challenges of complete debridement.

These findings highlight that combining advanced mechanical systems like the Gentle Brush with potent irrigants such as EDTA is the most effective approach for thorough canal cleaning. This integrated method overcomes the limitations of individual techniques and sets a new standard for enhancing clinical outcomes in root canal therapy. Future research should aim to optimize these methods further, evaluate their performance in varied anatomical scenarios, and explore their practical application to refine endodontic protocols.
